# Perceptions of Dietary mHealth Apps Among Adolescents: Scoping Review

**DOI:** 10.2196/91650

**Published:** 2026-10-20

**Authors:** Maria B Magro, Alysha L Deslippe, Trisha C Baluyot, Angela S Alberga, Tamara R Cohen

**Affiliations:** 1 Department of Health, Kinesiology and Clinical Exercise Physiology, Concordia University, Montreal, QC, Canada; 2 Food, Nutrition and Health, Faculty of Land and Food Systems, University of British Columbia, 218-2205 East Mall, Vancouver, BC, V6T 1Z4, Canada, 1-604-827-0362; 3 Healthy Starts, BC Children's Hospital Research Institute, Vancouver, BC, Canada; 4 Centre of Excellence in Adolescent Severe Obesity, Department of Adolescent Medicine, Montreal Children's Hospital, Montreal, QC, Canada; 5 Research Institute of the McGill University Health Research Center, McGill University, Montreal, QC, Canada

**Keywords:** adolescents, mobile health, mHealth, mobile apps, diet, nutrition, scoping review

## Abstract

**Background:**

Adolescents’ high engagement with smartphones creates opportunities for mobile health (mHealth) apps targeting dietary behaviors. However, despite the availability of diverse dietary mHealth apps, user engagement remains low. Existing quantitative literature has identified barriers and facilitators to app effectiveness in youth (aged 10-19 years) but has not fully captured the nuanced perceptions of adolescents (aged 12-19 years), who represent a unique life stage. Synthesizing similarities and differences in adolescents’ perceptions of what they value in dietary mHealth apps from qualitative studies may provide a more comprehensive understanding of app engagement and future app design.

**Objective:**

We aimed to synthesize adolescents’ perceptions of using diverse dietary mHealth apps.

**Methods:**

A scoping review was conducted according to the 5-stage framework by Arksey and O’Malley. Eligible studies were published in English and reported on adolescents (aged 12-19 years) with perceptions of dietary mHealth apps using qualitative methods. Apps that were not specific to diet, relied only on SMS text messaging, were delivered as part of a school-based intervention or telemedicine service, or were housed on a website or social media platform were excluded. Five evidence sources (PsycINFO, Embase, Medline, Web of Science, and CINAHL) were searched. Extracted data were charted by 1 researcher following a pilot test. Similarities and differences in adolescents’ perceptions were synthesized across studies using thematic analysis.

**Results:**

Of the 1195 search results, 1.2% (n=14) met the eligibility criteria. We identified six themes that represented adolescents’ perceptions of using dietary mHealth apps: (1) functionality and design, (2) content and depth, (3) social connection and support, (4) entertainment and gamification, (5) goal setting and motivation, and (6) perceived effectiveness. Overall, adolescents favorably perceived apps that were easy to navigate, fostered social connections, were personalizable, and were gamified. Apps that were felt to increase awareness of personal eating habits, often through tracking calories and foods consumed, and foster awareness of personal well-being were especially discussed in relation to perceived app effectiveness.

**Conclusions:**

The findings of this review provide an innovative synthesis of adolescents’ perceptions of diverse dietary mHealth apps, using qualitative data. Notably, dietary mHealth apps that were perceived as easy to navigate, tailored (eg, in terms of content), and gamified and those that included social engagement were perceived favorably, regardless of their dietary target and features. Apps that were perceived to improve adolescents’ current dietary awareness were particularly valued. Inclusion of these elements in dietary mHealth apps may promote greater engagement. App developers should work with adolescents to establish how specific apps can incorporate these elements into their design, including how best to track dietary habits and integrate AI into app features.

## Introduction

Adolescence is a critical period marked by rapid physical growth and cognitive development [1,2]. There is broad consensus across disciplines that promoting health-protective behaviors during this life stage, including adequate consumption of fruits and vegetables, can help reduce the risk of chronic disease in adulthood [1,2]. Despite this, evidence from around the world indicates that adolescents’ dietary behaviors often do not align with established recommendations [3-5]. For example, the literature from Canada and the United States reports that adolescents tend to consume diets high in sugar, fat, and sodium and frequently skip breakfast [3,4]. These patterns are consistent with findings from Europe and Oceania [6]. In contrast, current dietary guidelines emphasize the importance of consuming fruits and vegetables, whole grains, and protein-rich foods, while limiting foods that have added sodium, sugars, or saturated fats [7,8].

Similar misalignment between dietary recommendations and apparent fruit and vegetable consumption, fast food intake, and sugary carbonated beverage consumption has been observed in Thailand, Suriname, and Nepal [5]. Collectively, this body of evidence highlights that improving adolescents’ dietary habits remains an ongoing global public health priority [3-6].

In high-income countries, smartphones may offer a promising avenue to address the gap between adolescents’ dietary habits and expert dietary recommendations, given their widespread use within this population, with ownership rates frequently exceeding 90% [9,10]. Dietary interventions delivered through a mobile health (mHealth) platform (eg, apps) have shown significant improvements in dietary habits [11,12]. These interventions may facilitate behavior change by being highly accessible to adolescents [13-15] and often anonymous, thereby reducing barriers such as stigma and cost associated with accessing in-person support [15,16].

General mHealth app use has been associated with significant reductions in sugar-sweetened beverages [11,12,17] and energy-dense snacks [11,12]. Additionally, a systematic review of 14 digital interventions, including 9 targeting dietary outcomes among youth (aged 9-18 years), found improvements in fruit and vegetable intake in 3 studies [17]. However, excessive smartphone use (including use of apps) has also been associated with poorer dietary habits, including reduced fruit and vegetable intake and higher intake of fast food and sugar-sweetened beverages [18]. This dual potential highlights the importance of understanding variations in adolescents’ app experiences to identify those most likely to benefit from dietary mHealth interventions [11].

Literature evaluating mHealth app experiences has predominantly focused on identifying barriers and facilitators to app use and effectiveness [11]. A 2023 systematic review of 14 studies outlining youths’ (aged 10-19 years) experiences with 13 mHealth apps, 7 of which focused exclusively on diet, identified personalized messages and rewards as key facilitators of effectiveness [11]. Two additional systematic reviews published within the past 5 years, drawing on qualitative studies of youth (aged 4-18 years), reported similar findings, highlighting the value of personalization and rewards, along with simple interfaces, varied content, and inclusion of social support features [19,20].

However, these studies did not distinguish adolescents’ perceptions and experiences from those of younger children. This limitation is important, as adolescence is characterized by rapid physical growth and pubertal changes that influence appetite [21] and nutritional needs [22]. It is also a period of identity formation, shifting dietary motivation, and increasing eating outside the home [23,24]. Consequently, findings that aggregate broad age ranges may obscure adolescent-specific experiences with mHealth apps. More recent systematic reviews have not addressed this gap and instead focus on specific outcomes such as sustainable dietary habits (eg, reduction in meat consumption) [25,26]. Consequently, there remains limited understanding of adolescents’ perceptions of mHealth apps aimed at general healthy eating.

Poorer app experiences contribute to app disengagement, which may compromise intervention fidelity regardless of app quality [27]. Adolescents who do not fully engage with an app or discontinue use early are likely to be exposed to fewer of its key components and content, limiting its potential to influence dietary behaviors [27]. Given that low engagement is a persistent concern in mHealth research [11,18,28], with many users disengaging within the first month, a more nuanced understanding of adolescents’ perceptions of mHealth apps is needed. Such insights could inform app design and improve engagement, thereby enhancing the likelihood of meaningful dietary changes [29]. Existing evidence syntheses have largely focused on barriers and facilitators to app use and effectiveness, often emphasizing the inclusion or exclusion of features [11,19,20] and intervention components [25,26]. However, these approaches may overlook broader experiential factors that shape engagement. This study aimed to build on existing reviews by synthesizing adolescents’ perspectives on mHealth app use, identifying both similarities and differences in experiences.

## Methods

### Overview

We conducted a scoping review following Arksey and O’Malley’s [30] methodological framework. Details of our methods and the subsequent results are reported according to the PRISMA-ScR (Preferred Reporting Items for Systematic Reviews and Meta-Analyses Extension for Scoping Reviews) guidelines [31]. The PRISMA-ScR checklist can be found in Checklist 1.

### Protocol and Registration

The review protocol was created by the research team and uploaded to the OSF prior to the initiation of the search [32].

### Eligibility Criteria

To be eligible for this review, studies had to be published in English and report on adolescents (aged 12-19 years) and their engagement with an mHealth app related to diet and nutrition. This included studies that explored user perceptions of using these apps. We included qualitative and mixed methods studies that captured perceptions but only analyzed their qualitative data to increase our understanding of the nuances in reported adolescent perceptions that are not typically captured with quantitative data [33]. We excluded studies that reported on adults aged >19 years and children aged <12 years, as they do not experience the same behavioral, social, and biological changes that adolescents face [34]. Furthermore, literature reviews, scoping reviews, systematic reviews, and meta-analyses were not eligible for inclusion. Studies that reported on a school-based intervention, telemedicine services, online platforms, social media, and SMS text messaging systems were excluded to ensure that smartphone apps were the primary focus. Theses, dissertations, conference papers, protocols, and methodology papers were also excluded. There were no restrictions on the year of publication.

### Information Sources

In consultation with the research team and 2 trained research librarians, PsycINFO, Embase (Ovid), Medline (Ovid), Web of Science, and CINAHL (EBSCO) were selected as appropriate sources of information.

### Search

The search strategy was created in consultation with the university research librarian in 3 stages. First, the search terms were determined using the population, concept, and context framework [35]. Second, the research team identified 3 core papers that had recently been published and were considered critical to answering the research question based on the team’s field expertise in dietary behavior change. Finally, with the research librarian, a research assistant developed and tested search syntaxes in all of the identified sources of evidence until all core papers were captured in the searches. The final searches that captured those core papers were considered robust and used. The search strategy and syntax can be found in Table 1.

**Table 1.** Search strategy and example of syntax used.

Domains and inclusion		Exclusion	Example syntax^a^
Population			
Adolescents aged 12-19 years		Youth aged <12 years and >19 years	(MH “Adolescence”) or (adolescen* or teen* or youth or youths)
Concept			
Perceptions (barriers, facilitators, and experiences)		Telemedicine apps, website-based platforms, SMS text messaging and chat systems, and apps delivered through a social media platform (eg, Facebook) instead of their own unique platform	(MH “Health services accessibility”) or (experience* or view* or outlook* or barrier* or facilitator* or challenge* or opportunit* or access* or needs or concern* or perception* or perspectiv*)
Mobile health app		Telemedicine apps, website-based platforms, SMS text messaging and chat systems, and apps delivered through a social media platform (eg, Facebook) instead of their own unique platform	(MH “Mobile Applications”) or ((mobile or tele* or virtual or digital or mhealth or m-health or smartphone or cellular) N2 app*)
Dietary behaviors and/or nutrition		Telemedicine apps, website-based platforms, SMS text messaging and chat systems, and apps delivered through a social media platform (eg, Facebook) instead of their own unique platform	(MH “Eating behavior+” or (MH “Diet+” or (MH “Eating”) or (diet* or eat or eating or nutrition)
Context			
Apps available to the general adolescent public.		Apps delivered as part of a school-based intervention	No limits applied to avoid prematurely missing an article. Instead, criteria were applied during abstract screening.
Study design			
Report on adolescents’ perceptions using qualitative methods		Literature reviews, scoping reviews, systematic reviews, meta-analyses, dissertations, conference papers, protocols, and methodology papers	No limits applied to avoid prematurely missing an article. Instead, criteria were applied during abstract screening.
Language			
English		Non-English	No limits applied to avoid prematurely missing an article. Instead, criteria were applied during abstract screening.

a Example syntax shown for the Cumulative Index to Nursing and Allied Health Literature (CINAHL) database. All search syntax components (population and concept) were combined with the Boolean operator AND. Truncation (*), exploding the search to include the main heading and all narrower related terms (+), adjacency operators (N2; within 2 words), and controlled database vocabulary (MH: Medical Heading) were tailored to each respective database. The search syntax included terms related to nutrition apps that were delivered as part of a medical intervention (eg, MH “health services accessibility”). After the search, the research team amended the original inclusion criteria to exclude all nutrition apps delivered as part of a medical intervention (eg, telemedicine) to avoid capturing perceptions that were related to medical support and not the app itself (eg, a clinical care team). The decision was made prior to any abstract screening and applied across all originally returned articles.

### Selection of Sources of Evidence

All returned articles from the 5 searched databases were exported into Covidence (Veritas Health Innovation), a web-based reference management software system [36]. Duplicate records were automatically removed by Covidence software. Two research students then independently screened all titles and abstracts for eligible studies. Following this, full texts were reviewed by the same 2 research students. All screening (abstracts and full texts) was done individually, with discrepancies discussed and resolved with the project’s senior authors (ASA and TRC).

### Data Charting Process

A third research student who was not involved in screening (MBM) completed data extraction independently. MBM used Microsoft Excel (version 2408; Microsoft Corp) to manage the extracted data. Extracted data items were tentatively created prior to the data extraction phase based on previous reviews the research team had conducted. The final list was refined during a pilot extraction using 10% (n=3) of included studies.

### Data Items

Extracted data items from the 3 piloted studies encompassed publication information (year, journal, and author names), study information (location; delivery setting; design; research questions; hypotheses; methods of data collection, analysis, and recruitment; inclusion and exclusion criteria; study length; and stated limitations), participant characteristics (age and/or grade; total sample size; attrition rate; sex and/or gender; prevalence of clinical conditions; and ethnic, racial, and/or cultural background), app details (length of use, app description, stage of development, app name, targeted behaviors, measured outcomes, and app development framework), and app perceptions (barriers, facilitators, attitudes, beliefs, motivators, and suggestions for improvement). After initial extraction, the research team identified the most relevant data items based on the eligible studies. For example, studies did not test the efficacy or effectiveness of a specific dietary intervention; thus, data items such as “length of intervention” were not relevant.

### Critical Appraisal of Individual Sources of Evidence

We did not perform a critical appraisal of the included individual sources of evidence, as per the standard protocol of a scoping review [31].

### Synthesis of Results

We conducted a thematic analysis [37] to synthesize the extracted data on adolescent perceptions of using dietary mHealth apps. A research student (MBM) first extracted stated app perceptions from the 14 studies using the predetermined categories from the extraction template (eg, barriers). Next, MBM inductively coded similarities and differences in extracted data within a category regarding app perceptions across eligible studies to develop a codebook. Codes were added during the review of each subsequent study. Added codes were then compared with codes from previously reviewed studies in an iterative fashion. Codes were grouped together to inform subthemes based on similarities and differences in underlying meaning. Subthemes were then mapped to inform broader overarching themes. The development of the codebook and the mapping of the themes occurred through discussion among the broader research team (ALD, TCB, and ASA). A summary of all themes and their subthemes is presented in Table 2.

**Table 2.** Summary of identified themes and subthemes exploring adolescents’ perceptions of dietary mobile health (mHealth) apps.

Themes and subthemes	Sample quotes^a^
Functionality and design (app performance, display, and access)	
Accessibility	“…participants had problems using a type of mobile phone that they were not accustomed to [Android].” [38]
Ease of use	“Participants did not want to spend too long figuring out how to use the app.” [39]
Tracking	“Entering information on food consumed was difficult at times.” [40]
Visual appeal	“…wanted a brighter color theme.” [41]
Concept and depth (quality, depth, and delivery of nutritional information)	
Trusted feedback	“[Adolescents] appreciated getting feedback about their dietary intake in the…app.” [38]
Multicomponent	“Features that were suggested by adolescents included information about emotions, mood, and their impact on motivation.” [42]
Social connection and support (role of social features in influencing app use)	
Peer connection	“Peers…influenced young people to use apps and device through promoting understandings that they were ‘cool,’ ‘in trend,’ and/or ‘popular’.” [43]
Family interaction	“If you know people who always use it, you can ask them about it, they always recommend apps you should use.” [43]
Entertainment and gamification (importance of making apps fun via gamification)	
Tells a story	“They liked the storyline of the game and its mountains climbing theme.” [44]
Captures my attention	“I loved this video because of how lively it was! And I accept the mindful eating challenge!” [45]
Rewards	“High levels of enjoyment, especially earning points in the game, were reported.” [44]
Goal setting and motivation (emphasis on goal-setting as a way to motivate app use)	
Personalized	“[Participants] liked the idea of being able to set progressive challenges and their own goals.” [40]
Hold accountable	“If you accomplished all three goals very well, you’ll get a gold badge…If you didn’t accomplish any goals, you will not get a badge.” [46]
Perceived effectiveness (perceptions of what contribute to app effectiveness)	
Tracking eating habits	“I sometimes did not eat things because then I would have to record them.” [38]
Increased knowledge	“Importance of an app that provides them with educational information and manages individualized patient information.” [47]
Mind-body connection	“I tried eating my dinner last night mindfully. When I did, I was surprised at how much better my food tasted and how I ate less than [normal].” [45]

a Quotes were extracted from the respective article’s text or supplemental materials.

## Results

### Selection of Sources of Evidence

Initially, 1195 studies were identified from the databases. A total of 605 (51%) duplicate studies were removed by Covidence, and 590 (49%) were screened for eligibility. Of the screened studies, 1% (n=14) met the stated criteria. The flow diagram with a complete justification for exclusion can be found in Figure 1.

**Figure 1.**
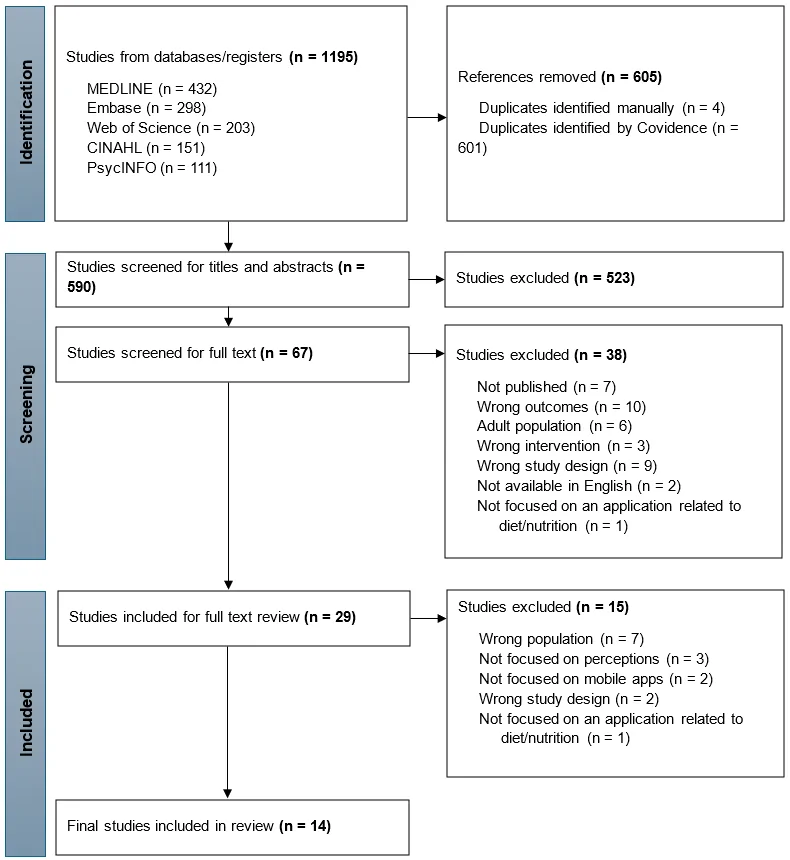
PRISMA-ScR (Preferred Reporting Items for Systematic Reviews and Meta-Analyses Extension for Scoping Reviews) flowchart.

### Characteristics of Sources of Evidence

A summary of extracted study characteristics is provided in Multimedia Appendix 1. Most studies were conducted in high-income countries in North America (4/14, 29%) [39,42,44,45] and Europe (n=6, 43%) [38,40,41,43,48,49], with 4 (29%) conducted outside of these regions, in the Dominican Republic, Indonesia, Korea, and Vietnam [46,47,50,51]. A total of 8 (57%) studies recruited adolescents from schools [38,40,41,43,46,49-51], 4 (29%) from clinical settings (eg, hospitals) [39,42,44,48], and 2 (14%) from community organizations [45,47]. Sample sizes ranged from 5 to 245 adolescents. Participants were between the ages of 12 and 19 years, with female subgroups ranging from 3 to 149 participants and male subgroups ranging from 0 to 106 participants. Only 6 (43%) papers were published before 2020 [38,42,43,45,48,50]. A total of 9 (64%) studies used mixed methods [38,40,41,43-45,48-50], whereas 5 (36%) used qualitative methods only [39,42,46,47,51].

Of the 14 described apps, 6 (43%) focused on diet and nutrition [38,40,41,46,50,51], while 4 (29%) other apps involved additional health behaviors such as physical activity [43-45,49]. Only 2 (14%) studies explored perceptions of adolescents living with a clinical condition (type 1 diabetes) [47,48], and 2 (14%) focused on weight management [39,42]. A total of 6 (43%) apps were in the pilot-testing stage [38,41,45,48-50], 3 (21%) were in development [40,44,46], 3 (21%) were available for download on the market [39,43,51], and 2 (14%) were described as a concept [42,47].

When it came to the use of behavioral theory to guide development, 1 (7%) study mentioned the use of integrated social cognitive theory, self-determination theory, and narrative transportation theory [44]. One other study used development frameworks such as the integrate, design, assess, and share framework (1/14, 7%) [40].

### Critical Appraisal Within Sources of Evidence

We did not perform a critical appraisal within sources of evidence of the included studies, as per the standard protocol of a scoping review [31].

### Synthesis of Results

We identified 6 themes to describe adolescents’ perceptions of using dietary mHealth apps that are outlined in the following sections. A summary of all identified themes with examples of the text extracted can be found in Table 2.

#### Theme 1: Functionality and Design

This theme addresses adolescents’ experiences with the technical performance and usability of dietary mHealth apps. Four subthemes captured adolescents’ perceptions of what hindered and supported their use of dietary mHealth apps (accessibility, ease of use, visual appeal, and tracking).

Adolescents spoke about the important role of creating apps that were accessible regardless of their device and personal context. In particular, this included the need for apps that could be used across software platforms and were accessible on their personal devices, not just those provided by a research team [48]:

…downloading [the app] to their own phones, they would continue to use the applications.

Additionally, adolescents expressed a need for apps that could evolve with their personal content, including having the ability to log diverse foods that they consumed and having access to food lists that were continually updated [51]:

…highlighted the importance of updating the list of beverages and drinks, as some kinds of drinks were not included in the database.

Motivation to use an app was discussed relative to its perceived ease of use and visual appeal. Adolescents spoke about the value of apps that were simple, responsive, readable, and similar to apps they already integrated into their everyday lives [39]:

[Participants] used popular apps such as Facebook, Instagram, and Snapchat and specifically reported that they preferred certain weight management apps because it reminded them of those popular app.

Esthetic components of apps, including design choices about color, font size, and display layout, were also highlighted as important characteristics [51]:

I don’t think it captures the user’s interest much [without images].

Most apps explored in the 14 studies included some form of dietary tracking (eg, calorie counting). Importantly, adolescents often valued having such features within an app, but recognized nuances associated with these features that influenced having a positive app experience. For some, tracking dietary data led to concerns about privacy, was burdensome, and created confusion when errors appeared [38]:

[Participants’] views that the recording of dietary intake was burdensome.

#### Theme 2: Content and Depth

This theme reflected adolescents’ views on the quality, depth, and delivery of nutritional information and was reflected in 2 subthemes (trusted feedback and multicomponent apps).

Adolescents valued apps that they felt provided accurate and trusted dietary and nutrition information. This included helping with reading food labels [39], understanding their personal nutrition needs, and providing space to receive feedback from experts:

[Adolescents] liked taking pictures of barcodes on food items with their telephone so that they could identify the nutritional information regarding foods that they were considering eating.

In addition to receiving trusted dietary and nutrition advice, adolescents also emphasized how apps that delivered content related to other behaviors, such as physical activity [47] and mental health, were desirable:

Participants suggested the possibility of having an exercise section in the app.

#### Theme 3: Social Connection and Support

This theme emphasizes the role of social features in influencing adolescents’ engagement with dietary mHealth apps. Two subthemes (peer connection and family interaction) were identified.

Adolescents spoke about the value of having spaces within the dietary mHealth apps to communicate with their peers. This was valued as a tool to discuss behavior change strategies, gain encouragement when goals were achieved [42], and socialize irrespective of the topic:

Encouragement and coping support were also discussed as potential benefits to a social support feature, which would allow for adolescents to share encouraging messages with peers and to seek out or offer advice.

Adolescents valued apps that included options to compete or play in-app games with peers [40], and adolescents recognized the added motivational value that could come from having an app and its messaging seem more desirable when their peers also used it:

Participants suggested that they could be more engaged with the game if they could interact with other players.

Family support was also recognized as a way to promote app use through normalization of its use. In contrast to peer-based support, adolescents spoke about how dietary mHealth app use could contribute to disruption of family routines, depending on what an app asked an adolescent to do [46]:

Inconvenience for family members in waiting for the process of taking pictures completely before starting to eat their meals.

#### Theme 4: Entertainment and Gamification

This theme captured adolescents’ perceptions of the importance of making apps fun via gamification aspects and specifically was broken down into 3 subthemes (tell me a story, capture my attention, and rewards).

Adolescents appreciated apps that used a storyline to deliver relevant dietary information and/or behavior change strategies [40]:

Positive about the main idea of the game, mainly because of the novelty of the topic and the setting of the game.

Use of flashy and attention-grabbing digital content such as videos and images was also desired [49]:

Perhaps it would be good to have images? I don’t think it captures the user’s interest much [without images].

Finally, the use of rewards helped make engagement fun [40]:

Being rewarded with medals that were visible to friends would be seen in a positive way as being motivating to them.

#### Theme 5: Goal Setting and Motivation

This theme reflects adolescents’ perspectives that goal setting could act as a powerful feature to motivate app use and is separated into 2 subthemes (personalized and hold accountable).

Being able to set and monitor personal dietary goals was expressed positively by adolescents across the studies [46]:

Enjoyed seeing daily statistics for individual dietary goals.

Use of goal setting as a feature to motivate dietary behavior change was also talked about in conjunction with the use of reminders [39] and rewards for completing a goal:

Self-monitoring features, such as having reminders, alerts, or notifications to keep them on track with their healthy behavioral goals was a highly preferred feature.

#### Theme 6: Perceived Effectiveness

This theme summarizes adolescents’ perceptions of specific app components that they attributed to the effectiveness of a dietary mHealth app in altering their behaviors. Specifically, tracking eating habits, increased knowledge, and mind-body awareness were 3 identified subthemes.

Tracking eating habits was favorably viewed as helping adolescents self-monitor their habits and shift their behaviors. This was discussed through the act of recording nutrients and meals consumed, as well as by tracking what was eaten in relation to other aspects of health and well-being outcomes such as monitoring diabetes [47]:

The new app could assist them in managing their disease [such as] assistance with carbohydrate counting and food identification, better decisions on food choices using a list of glycemic indexes, and assistance with daily management of the disease.

Perceptions of increased knowledge, whether related to nutrient needs or to related aspects of health, were further attributed to increased desire to make dietary changes [45]:

Better visual understanding of the cornerstones of diabetes self-management: food intake,....

Finally, observing changes in feelings of well-being [45] further helped adolescents feel that using a dietary mHealth app was valuable:

[Adolescents who perceived] the app to be very helpful and enjoyable and reported feeling more relaxed, focused, and peaceful.

## Discussion

### Summary of Evidence

This scoping review synthesized evidence on adolescents’ perceptions of dietary mHealth apps across 14 studies. Importantly, we focused on adolescents aged 12 to 19 years, in contrast to the broader age ranges of prior reviews that also focused on youth (aged 10-19 years) [11,19,20]. From the evidence synthesis, six themes were identified that captured adolescents’ perspectives: (1) functionality and design, (2) content and depth, (3) social connection and support, (4) entertainment and gamification, (5) goal setting and motivation, and (6) perceived effectiveness. Our findings build upon prior reviews that have focused on presenting barriers and facilitators of app features [11,19,20,25,26] to provide a more nuanced perspective that can be used across diverse dietary mHealth apps.

Overall, adolescents value dietary mHealth apps that are simple, fun, and raise awareness of their current habits. Technical ease [19,20], esthetic appeal [19,20], gamification [11,19,20], avenues of social support [20,25], and use of goal setting [19,20,25,26] have all been reported in prior literature as essential facilitators to dietary mHealth app engagement, matching key themes that arose in this scoping review. Importantly, apps that were perceived to be more effective by adolescents specifically included dietary tracking and increased knowledge that moved beyond just nutrition facts, opening up novel avenues for research to consider.

### Calorie Counting, Dietary Tracking, and Behavior Change Promotion

Tracking eating habits emerged as a critical subtheme influencing adolescents’ perceived dietary changes. This included approaches such as calorie counting and tracking different types of foods consumed. Such dietary self-monitoring is a common feature of many dietary mHealth apps [52]; a 2021 systematic review found that 20 out of 28 included nutrition apps incorporated calorie counters [52]. The review identified goal setting, personalization, and dietary self-monitoring [52] as key facilitators of app engagement.

Within behavioral medicine, calorie counting is categorized as a behavior change technique (BCT), reflecting dietary self-monitoring [53]. Evidence supports its effectiveness: a meta-regression of 122 diet and physical activity interventions in adults found that self-monitoring accounted for the greatest variability in outcomes, with stronger effects observed when combined with other techniques [54]. These findings align with the present results, suggesting that dietary self-monitoring through strategies such as calorie counting may support behavior change. However, recent literature has raised concerns about calorie counting as a dietary strategy. It can be burdensome, requires a high degree of nutrition literacy [55], and may promote an overemphasis on numerical targets rather than internal cues of hunger and satiety (eg, intuitive eating) [56].

Many countries have adapted national dietary guidelines that use plate-based eating (eg, MyPlate) [56]. These approaches guide intake using proportions of macronutrients (eg, protein) and broader food groups (eg, grains), offering a more visual and intuitive framework for food choices. A Canadian study examining the perspectives of registered dietitians (n=44) and the general public (n=52) found that plate-based approaches were valued for promoting dietary self-awareness through simple visual representations of foods on a plate [55]. Despite this, few mHealth apps have adopted plate-based approaches.

Given that adolescents in this scoping review, as well as prior studies [19,20], report valuing simple and visually engaging app content, plate-based eating may represent a promising dietary self-monitoring strategy in digital content. Beyond simplicity, this approach connects how they feel with what they eat by shifting focus away from numerical targets. This aligns with principles of intuitive eating that emphasize internal cues such as hunger and satiety [56] and are associated with lower odds of poor mental well-being (eg, high depressive symptoms and low self-esteem), body dissatisfaction, and negative weight control behaviors (eg, skipping meals) among adolescents in longitudinal research (n=1491) [57]. Participants in this scoping review also expressed interest in app content linking mental health and dietary behaviors (eg, mind-body awareness subtheme). Taken together, these findings suggest that incorporating plate-based eating into mHealth apps warrants further exploration as a developmentally appropriate and potentially supportive dietary self-monitoring strategy.

### Peers and Family Systems as Enactors of Social Support

In this scoping review, social interaction emerged as a key component of valued dietary mHealth apps, consistent with previous reviews [20,25,52]. Importantly, peers and the family unit were identified as particularly important sources of social support. In contrast to prior research, our findings did not highlight a perceived need for the involvement of clinical professionals, suggesting that adolescents may prioritize informal support networks over formal health care input in the context of app use.

A 2023 scoping review of 43 studies evaluating dietary mHealth apps among individuals living with chronic disease (n=256,430; eg, cancer) found that social features connecting users with clinicians or health coaches were key to sustaining behavior change [58]. In contrast, interactions with health professionals were not a central focus for adolescents included in our scoping review. This divergence may reflect differences in health status; adolescents managing complex or chronic conditions may face a greater burden, increasing the perceived value of clinical support.

This interpretation is consistent with findings from studies included in our review, where adolescents valued app content that linked dietary behaviors to disease management (eg, effects on insulin levels in diabetes). As such, these findings suggest that preferences for social support features may vary depending on the health context. Future research is needed to better understand when and for whom different forms of support, such as health coaches or clinical professionals, are most relevant and effective for adolescents using mHealth apps.

In the growing world of AI, it is also important to clarify adolescents’ perspectives on AI as a social support feature (eg, a chatbot). A scoping review that explored youths’ (aged 10-19 years) acceptability of chatbots in nutrition and physical activity apps found that, in 2 of the 5 reviewed studies, adolescents expressed satisfaction with a chatbot [59]. Interestingly, when the authors discussed the findings on chatbots with a youth advisory panel, youth brought forward additional concerns surrounding the possibility of spreading misinformation through chatbots and data privacy [59]. Although adolescents in our scoping review did not explicitly discuss chatbots, they did highlight privacy concerns with sharing data in an app. This highlights the need to further explore the role of AI within dietary mHealth apps as a source of information or dietary consultant and to understand how adolescents perceive the use of AI within dietary apps as a potential social support feature in greater depth.

### Multicomponent Apps

In this scoping review, adolescents expressed an interest in the inclusion of physical activity and mental health support. Previous literature has replicated this finding, with many apps integrating both behaviors alongside each other [11,19,59]. Importantly, our findings highlighted adolescents’ perceptions that mental health information and features that foster reflection on emotions and mood were valuable. Although numerous apps exist that target aspects of mental health and well-being [60], such apps are often stand-alone apps that do not encompass dietary aspects or are developed for individuals living with eating disorders [61]. This raises questions as to the extent to which mHealth apps should target more holistic approaches to health as a facet of physical and mental well-being in 1 place, and if such an approach would have benefits for adolescents’ behavioral outcomes.

### Limitations

Most included studies in this review were conducted in North America and Europe, which limits the generalizability of our findings to diverse cultural contexts and countries where smartphone use is lower. As recent literature has reported lower smartphone use among adolescents from low- to middle-income countries, especially among female adolescents [62], our findings may not be relevant to all adolescents. We further did not evaluate any objective measures of app efficacy and effectiveness. This means that we are not able to draw conclusions about associations between changes in behaviors and our findings. Furthermore, data charting and synthesis were conducted by a single reviewer, which may have introduced bias in study selection and interpretation. To help mitigate this risk, the research team collaboratively developed the data extraction form and resolved ambiguities through consensus. We also did not have the ability to probe for more specific perceptions of app use across unique devices and contexts. It is possible that the type of device a user owns (eg, Android vs iOS) or context (eg, delivered through a school vs clinical site) may impact adolescents’ perceptions of the apps, and these nuances are not captured here. Finally, given the rapid evolution of adolescent smartphone use and mHealth app development, our findings may not reflect the most recent innovations that are on the market today. Future reviews should consider using living methods to allow continual updating of information as it becomes available.

### Conclusions

This scoping review provides comprehensive and novel insights into adolescents’ perceptions of dietary mHealth apps across a range of dietary mHealth apps—a group whose perceptions have not been explored in depth. Adolescents expressed an interest in apps that are easy to navigate, foster social connections, can be personalized, and have gamified elements. In particular, apps that were perceived to improve awareness of one’s dietary habits through tracking and connections to aspects of well-being were desired. Future work is needed to explore how dietary tracking can be best facilitated and the extent to which AI has a role within adolescent-focused dietary mHealth apps.

## Supplementary material

10.2196/91650 Multimedia Appendix 1 Characteristics of sources.

10.2196/91650 Checklist 1 PRISMA-ScR checklist.

## Abbreviations

BCT: behavior change technique
mHealth: mobile health
PRISMA-ScR: Preferred Reporting Items for Systematic Reviews and Meta-Analyses Extension for Scoping Reviews
