# Supplementary material for: Perceptions of Dietary mHealth Apps Among Adolescents: Scoping Review
Source: JMIR Pediatr Parent. 2026 Oct 20;9:e91650. doi: 10.2196/91650 (PMC13683992; doi:10.2196/91650)
Supplement: Supplementary file 1 [file pediatrics-v9-e91650-s001.docx]

Extracted study characteristics from 14 studies exploring adolescents’ perceptions of dietary mHealth apps.

| **Author, year** | **Country, setting** | **Study design** | **Age** | **Sample size (n; sex)** | **Aim of study** | **Data collection** | **Name of app, stage of app** | **Purpose of app** |
| --- | --- | --- | --- | --- | --- | --- | --- | --- |
| Braga et al., 2022 [49] | Vietnam, high school (public; urban) | Qualitative | 15-17-year-olds | 32 (32 female, 0 male) | To analyze acceptability, usability and likability of app using food recognition | Focus group discussions, semi structured questionnaires, field notes and observations | Food Recognition Assistance and Nudging Insights (FRANI) app, in development | To track and motivate healthier food choices through a gamified app with individual and team-based goals |
| Froisland et al., 2012 [44] | Norway, pediatric clinics | Mixed method | 13-19-year-olds with type 1 diabetes | 12 (7 female, 5 male) | To examine how apps can be used for type 1 diabetes management and explore adolescents’ experiences with mobile apps for diabetes management | Interviews, field notes, semi structured interviews, HbA1c levels, SUS questionnaire, 27-item diabetes questionnaire | Diamob, pilot testing | To evaluate carbohydrate and insulin dosages through documentation of food |
| Fuemmeler et al., 2020 [39] | USA, Pediatric clinics (oncology) | Mixed method | 12-17-year-olds with cancer | 16 (6 female, 9 male) | To describe feasibility and development of app in promoting a healthy diet and physical activity | Surveys, height and weight, interview, self-report food frequency questionnaire (FFQ), PACE Adolescent Psychosocial Measures, Transport Narrative Questionnaire | Mila Blooms, in development | To promote changes in dietary patterns by reducing intake of unhealthy foods and sugary beverages; promote physical activity |
| Goodyear et al., 2019 [43] | United Kingdom, schools (private, state, academies, faith) | Mixed method | 13-18-year-olds | 245 (139 female, 106 male) | To understand adolescents’ perspectives on the type and reason for health app usage | Participatory class activities, group tasks, questionnaires, semi structured interviews | MyFitness Pal, 7-minute workout, Under Armour, Fitbit, Pokémon Go,  On the market | N/A |
| Langlet et al., 2020 [45] | Sweden, school | Mixed method | Students with mean age (SD) = 16.8(0.7) years | 26 (17 female, 7 male) | To assess acceptability, usability, and constraints of app for dietary behaviours | Self-reported eating events, eating behaviour data through sensors, SUS question-naire, custom behaviour change and comfort questionnaire | SPLENDID app, pilot testing | To self-monitor dietary habits and eating behaviours |
| Lee et al., 2017 [50] | Korea, high schools | Mixed method | 16-18-year-olds | 33 (24 female, 9 male) | To assess usefulness of app for monitoring dietary intake in adolescents | Age, height, weight and weight control, physical activity, dietary habits and supplements, 24-hour recalls, feasibility questionnaire | Diet-A, pilot testing | To record dietary intake with provision of real-time feedback regarding nutrient, disease risk, and food recommendations |
| Martin et al., 2020 [46] | Spain, Italy, England & Scotland, schools (urban, rural) | Mixed method | 13-16-year-olds | 74 (N/A) | To describe co-design process and findings of app components prior to feasibility and pilot testing | Focus group sessions, participant agreement, SUS scale, brief use of app questionnaire, survey on game use | PEGASO Fit for Future (F4F), in development | To track eating habits and provide feedback and suggestions for improving dietary habits (e.g., increased fruit and vegetable consumption, healthy snacking and decreased fast food intake and breakfast skipping) |
| Moore et al., 2023 [51] | Dominican Republic, nonprofit institution | Qualitative | 14-18-year-olds with type 1 diabetes | 23 (9 female, 14 male) | To examine preferences for content, functionality, and design of app for type 1 diabetes management | Structured phone interviews, questionnaire, socio-demographic data | N/A, concept | To count carbohydrate for management of type 1 diabetes |
| Müssener et al., 2020 [47] | Sweden, high schools | Mixed method | 15-18-year-olds | 5 (3 female, 2 male) | To examine usability of app direct toward risky health behaviours | Usability test through goal-oriented tasks by targeted end users, heuristic evaluation through think-aloud method, SUS questionnaire | LIFE4YOUth, pilot testing | To promote eating healthy, physical activity, smoking cessation, and non-risky drinking by providing health behaviour information, behaviour change techniques, and activities |
| Rahmah et al., 2020 [52] | Indonesia, high school | Qualitative | Individuals in 8^th^ grade | 38 (38 female, 0 male) | To evaluate acceptance of app and observe differences between app-based and paper-based recording of fluid intake | Focus group discussions, in-depth interviews, audio recordings, observations, note-taking | My Fluid Diary, on the market | To self-monitor daily fluid intake from beverages |
| Rivera et al., 2018 [40] | Canada, pediatric hospitals | Qualitative | 12-18-year-olds with obesity and with disabilities | 19 (13 female, 6 male) | To perform user-centered needs assessment for app design targeting weight and health management | Demographic questionnaire, health questionnaire, focus group discussion (semi structured), observations, field notes | N/A, prospective concept | To provide weight and health management for adolescents with severe obesity and disabilities |
| San Giovanni et al., 2021 [41] | USA, pediatric research network | Qualitative | 13-17-year-olds with obesity | 14 (8 female, 6 male) | To explore app feature preferences, engagement and discouragement regarding weight management | Semi structured interviews, audio recordings, performance tasks | N/A, on the market | To provide weight management |
| Svensson et al., 2016 [48] | Sweden, schools | Mixed method | 14-16-year-olds | 92 (54 female, 38 male) | To explore user experience of app and factors affecting dietary intake record | Mobile dietary assessment, focus group interviews, in-app questionnaire, total energy expenditure, weight, height, self-report questionnaire, semi structured interview | N/A, pilot testing | To record dietary intake of food and beverages consumed |
| Turner & Hingle, 2017 [42] | USA, community organizations, online | Mixed method | 14-18-year-olds | 15 (7 female, 8 male) | To assess acceptability, feasibility and usefulness of app regarding mindfulness | Patient health questionnaire (PHQ-4), eating attitudes test (EAT-26), weight, height, in-app survey, poststudy survey | b@Ease Mindfulness app, pilot testing | To increase awareness of hunger and satiety cues, and sensory components of eating through guided mind-fulness techniques |
